# Studying Pathogenetic Contribution of a Variant of Unknown Significance, p.M659I (c.1977G > A) in MYH7, to the Development of Hypertrophic Cardiomyopathy Using CRISPR/Cas9-Engineered Isogenic Induced Pluripotent Stem Cells

**DOI:** 10.3390/ijms25168695

**Published:** 2024-08-09

**Authors:** Sophia V. Pavlova, Angelina E. Shulgina, Suren M. Zakian, Elena V. Dementyeva

**Affiliations:** 1 Federal Research Centre Institute of Cytology and Genetics, Siberian Branch of the Russian Academy of Sciences, 630090 Novosibirsk, Russia; shulginaae@bionet.nsc.ru (A.E.S.); zakian@bionet.nsc.ru (S.M.Z.); dementyeva@bionet.nsc.ru (E.V.D.); 2 Institute of Chemical Biology and Fundamental Medicine, Siberian Branch of the Russian Academy of Sciences, 630090 Novosibirsk, Russia

**Keywords:** hypertrophic cardiomyopathy, variants of unknown significance, CRISPR/Cas9, induced pluripotent stem cells, cardiomyocyte

## Abstract

Hypertrophic cardiomyopathy (HCM) is a cardiovascular pathology that is caused by variants in genes encoding sarcomere-associated proteins. However, the clinical significance of numerous variants in HCM-associated genes is still unknown. CRISPR/Cas9 is a tool of nucleotide sequence editing that allows for the unraveling of different biological tasks. In this study, introducing a mutation with CRISPR/Cas9 into induced pluripotent stem cells (iPSCs) of a healthy donor and the directed differentiation of the isogenic iPSC lines into cardiomyocytes were used to assess the pathogenicity of a variant of unknown significance, p.M659I (c.1977G > A) in *MYH7*, which was found previously in an HCM patient. Using two single-stranded donor oligonucleotides with and without the p.M659I (c.1977G > A) mutation, together with CRISPR/Cas9, an iPSC line heterozygous at the p.M659I (c.1977G > A) variant in *MYH7* was generated. No CRISPR/Cas9 off-target activity was observed. The iPSC line with the introduced p.M659I (c.1977G > A) mutation in *MYH7* retained its pluripotent state and normal karyotype. Compared to the isogenic control, cardiomyocytes derived from the iPSCs with the introduced p.M659I (c.1977G > A) mutation in *MYH7* recapitulated known HCM features: enlarged size, elevated diastolic calcium level, changes in the expression of HCM-related genes, and disrupted energy metabolism. These findings indicate the pathogenicity of the variant.

## 1. Introduction

Hypertrophic cardiomyopathy (HCM) is one of the most common cardiovascular diseases. It affects 1 of 500 people in the general population. HCM is characterized by thickened left ventricle walls and interventricular septum, diastolic dysfunction, progressive heart failure, and a high risk of arrhythmia and sudden death [1]. Up to 60% of HCM cases are inherited [2]. Most HCM-causing variants were identified in genes encoding sarcomeric proteins that belong to the contractile apparatus of cardiomyocytes. About 80% of inherited HCM cases are accounted for by variants in MYH7 and MYBPC3 that encode β-myosin heavy chain and myosin-binding protein C, respectively. More than 12% of cases are due to variants in *TNNT2*, *TNNI3*, and *TPM1* that encode cardiac troponinT, cardiac troponin I, and α-tropomyosin. In rare cases, HCM-causing variants were found in genes encoding other contractile sarcomeric proteins (*ACTC1*, *MYL2*, *MYL3*, *TNNC1*), Z-disc proteins (*ACTN2*, *ANKRD1*, *CSRP3*, *FLNC*, *LDB3*, *MYOZ2*, *NEXN*, *TCAP*, *VCL*), sarcomere-associated proteins (*DES*, *FHL1*), as well as proteins involved in the regulation of calcium homeostasis (*CALR3*, *CASQ2*, *JPH2*, *PLN*) [3,4]. Nevertheless, pathogenetic mechanisms underlying HCM are still poorly understood. A significant progress in HCM studying has been made using generation of induced pluripotent stem cells (iPSCs) from HCM patients carrying pathogenic variants and subsequent differentiation of the iPSCs into cardiomyocytes. The approach simplified the obtainment of cardiomyocytes with HCM-associated mutations and allowed us to shed more light on the mechanisms of disease development at the molecular and cellular levels [5,6,7,8,9,10].

CRISPR/Cas9 is a unique tool of genome editing with multiple applications in fundamental biology and biomedicine. A combination of CRISPR/Cas9 and the iPSC technology opens new prospects in studying cardiovascular diseases, including HCM [11,12,13]. The technique allows for the generation of isogenic iPSC lines that share genetic backgrounds and differ only by the presence/absence of a certain mutation. The differentiation of isogenic iPSC lines into cardiomyocytes and the comparison of properties of the cardiomyocytes make it possible to examine the net impact of the mutation on disease development. Using CRISPR/Cas9, several pathogenic variants were introduced into iPSCs to examine HCM molecular mechanisms [14,15,16,17,18]. Another way of applying CRISPR/Cas9 is correcting a pathogenic mutation in patient-specific iPSCs to generate isogenic control [19,20]. In addition, generating isogenic iPSCs with CRISPR/Cas9 is used for checking the pathogenicity of variants of unknown significance. The technology has been already used to determine the clinical significance of a number of genetic variants for HCM development [21,22]. This task seems to be very relevant because the implementation of next-generation sequencing methods in clinical practice has resulted in the identification of novel variants and genes associated with cardiovascular diseases. The clinical significance of more than 40% of the variants remains unknown [11].

A 38-year-old male patient diagnosed with obstructive HCM was previously found in a genetic screening of HCM patients [23]. The patient was characterized by asymmetric hypertrophy of the left ventricle, thickened interventricular septum (29–30 mm), congestive heart failure (NYHA Class II–III), moderate mitral valve regurgitation, an enlargement of both atria, SAM (systolic anterior motion) syndrome, and arterial hypertension. An analysis of the patient’s clinical exome revealed a heterozygous p.M659I (c.1977G > A) variant in *MYH7* (rs1241603111, MIM *160760 for the gene, MIM #192600 for *MYH7*-associated HCM). The substitution is localized to a functionally important actin-binding region of the myosin motor domain. The variant is absent in gnomAD (https://gnomad.broadinstitute.org/, accessed on 14 July 2024), and its minor allele frequency is currently unknown. It was found in HCM patients in a few studies [24,25]. The p.M659I (c.1977G > A) variant in *MYH7* is supposed to be pathogenic based on the data of an in silico analysis (Table 1, [23]) and AlphaMissense prediction [26]. However, the substitution is classified as a variant of uncertain significance in ClinVar, and its role in HCM development needs to be clarified.

In this study, we tried to determine the pathogenicity of the p.M659I (c.1977G > A) variant in *MYH7*. The mutation was introduced into the iPSCs of a healthy donor with CRISPR/Cas9 and single-stranded donor oligonucleotides. A modification of the approach was developed to generate iPSCs heterozygous at the substitution. iPSC-derived cardiomyocytes with the p.M659I (c.1977G > A) mutation introduced with CRISPR/Cas9 in *MYH7* were shown to display several HCM features, such as an enlarged cardiomyocyte size, elevated diastolic calcium level, changes in the expression of several HCM-related genes, and disrupted energy metabolism, which supports population and in silico data on the pathogenicity of the variant.

## 2. Results

### 2.1. Introducing p.M659I (c.1977G > A) Mutation with CRISPR/Cas9 in MYH7 of Healthy Donor iPSCs

**Figure 1:**
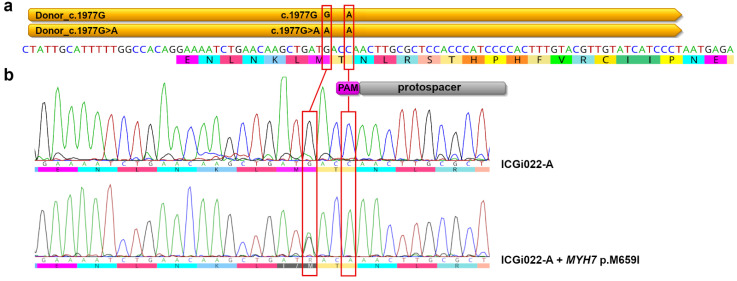
Introducing p.M659I (c.1977G > A) variant in *MYH7.* (**a**) Design of single-guide RNA and single-stranded donor oligonucleotides for *MYH7* editing. Nucleotide sequence of a part of *MYH7* Intron 17 and Exon 18 is provided. The localization of protospacer for the single-guide RNA, PAM, and the single-stranded donor oligonucleotides is shown in grey, magenta, and yellow, respectively. Positions of the target c.1977G > A substitution and synonymous substitution in PAM are indicated with red rectangles. (**b**) One iPSC clone heterozygous at p.M659I (c.1977G > A) mutation in *MYH7* was generated (ICGi022-A + *MYH7* p.M659I). Nucleotide sequence of the same region in the ICGi022-A iPSC line used for *MYH7* editing is provided for comparison. Positions of the target c.1977G > A substitution and synonymous substitution in PAM are indicated with red rectangles.

The p.M659I (c.1977G > A) mutation was introduced with CRISPR/Cas9 and single-stranded donor oligonucleotides in *MYH7* Exon 18 of iPSCs of the ICGi022-A line previously derived from the healthy donor [27]. CRISPR/Cas9 was delivered to the cells in the form of ribonucleoprotein complexes of single-guide RNA and Cas9_NLS. As the patient was a heterozygous carrier of the variant, we decided to use a combination of two single-stranded donor oligonucleotides for generating iPSCs heterozygous at the substitution. One donor oligonucleotide contained the p.M659I (c.1977G > A) mutation, and the other corresponded to the nucleotide sequence of *MYH7* Exon 18 of the healthy donor. Both donor oligonucleotides also comprised a synonymous substitution that modified PAM (Protospacer Adjacent Motif) sequence to protect *MYH7* alleles from repetitive CRISPR/Cas9 editing (Figure 1a).

Seventy iPSC clones have been generated after the iPSC electroporation with CRISPR/Cas9 and the single-stranded donor oligonucleotides. Non-homologous end joining occurred in 41 (58.57%) of the iPSC clones. Double-strand breaks were repaired via homologous recombination in six (8.57%) of the iPSC clones. In two of the iPSC clones with homologous recombination, only the donor oligonucleotide that corresponded to the nucleotide sequence of *MYH7* Exon 18 of the healthy donor was used. The target c.1977G > A substitution was detected in four iPSC clones with homologous recombination. However, in three iPSC clones, the second allele contained indels. Thus, only one iPSC line heterozygous at the p.M659I (c.1977G > A) mutation in *MYH7* was generated as a result of using both types of donor oligonucleotides for double-strand break reparation, which was confirmed by homozygous synonymous substitution in PAM (Figure 1b). No CRISPR/Cas9 off-target activity was found after sequencing the top-five CRISPR/Cas9 off-target sites in the iPSC line with the introduced p.M659I (c.1977G > A) mutation in *MYH7* and the original ICGi022-A line (Figure S1). The CRISPR/Cas9 off-target sites were predicted using IDT (https://www.idtdna.com/, accessed on 18 November 2022).

**Figure 2:**
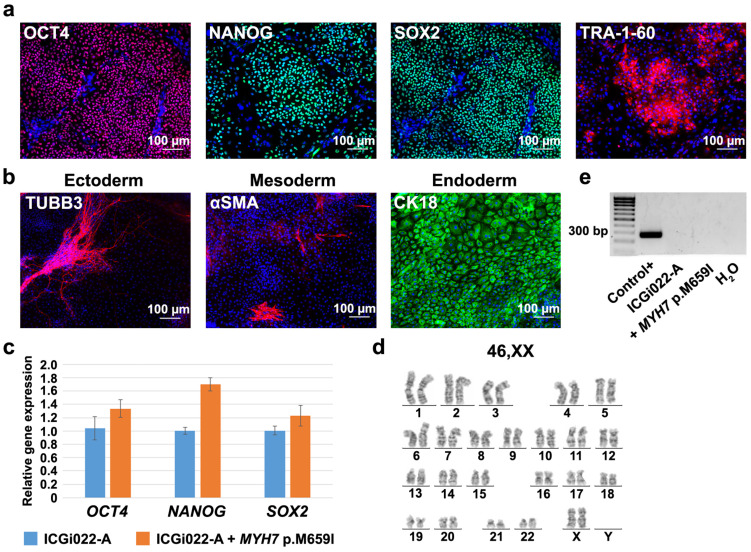
Characteristics of the iPSC line with introduced p.M659I (c.1977G > A) mutation in *MYH7*. (**a**) Positive expression of pluripotent state markers: the OCT4, NANOG, and SOX2 transcription factors and TRA-1-60 surface antigen in the iPSC line. Scale bar—100 µm. (**b**) The iPSC line can be spontaneously differentiated into derivatives of three germ layers: ectoderm (TUBB3, βIII-tubulin), mesoderm (αSMA, smooth muscle α-actin), and endoderm (CK18, cytokeratin 18). Scale bar—100 µm. (**c**) Expression level of *OCT4*, *NANOG*, and *SOX2* in the iPSC line with introduced p.M659I (c.1977G > A) mutation in *MYH7* (ICGi022-A + *MYH7* p.M659I) is comparable to that in the ICGi022-A iPSC line used for *MYH7* editing. (**d**) The iPSC line retains normal karyotype, 46,XX. (**e**) The iPSC line with introduced p.M659I (c.1977G > A) mutation in *MYH7* (+ *MYH7* p.M659I) is not contaminated with mycoplasma. Control +, positive control for mycoplasma contamination.

The iPSC line with the p.M659I (c.1977G > A) mutation, which was introduced with CRISPR/Cas9 in *MYH7*, retained pluripotent properties: expressed pluripotency markers, such as the OCT4, NANOG, SOX2 transcription factors and TRA-1-60 surface antigen, and gave rise to derivatives of three germ layers during spontaneous differentiation in embryoid bodies (Figure 2a,b). The expression level of *OCT4*, *NANOG*, and *SOX2* in the iPSC line was comparable to that in the original ICGi022-A line used for *MYH7* editing (Figure 2c). The iPSC line with the introduced p.M659I (c.1977G > A) mutation in *MYH7* also had a normal karyotype (46,XX (Figure 2d)) and was negative for mycoplasma contamination (Figure 2e).

### 2.2. Cardiomyocytes Derived from iPSCs with Introduced p.M659I (c.1977G > A) Mutation in MYH7 Demonstrate HCM Features

To study the impact of the p.M659I (c.1977G > A) variant in *MYH7* on HCM development, we differentiated the iPSC line with the introduced p.M659I (c.1977G > A) mutation in *MYH7*, an iPSC line from the patient with the variant, ICGi019-B [28], and two iPSC lines from healthy donors [27] (including the original ICGi022-A line) into cardiomyocytes. A protocol based on modulating the Wnt signaling pathway with the GSK3β protein kinase inhibitor (CHIR99021) and a Wnt inhibitor (IWP2 or Wnt-C59) was used to generate cardiomyocytes. The cardiomyocytes were purified via metabolic selection in a glucose-free medium supplemented with bovine serum albumin, ascorbic acid, and sodium DL-lactate [29].

**Figure 3:**
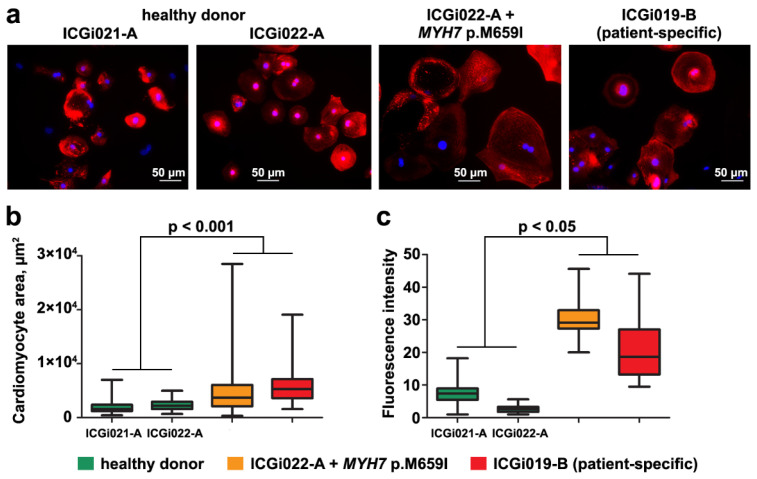
Cardiomyocytes derived from the iPSC line with introduced p.M659I (c.1977G > A) mutation in *MYH7* have enlarged size and elevated diastolic calcium level. (**a**) Examples of immunofluorescence staining of cardiomyocytes derived from two iPSC lines of healthy donors (ICGi021-A and ICGi022-A), the iPSC line with introduced p.M659I (c.1977G > A) mutation in *MYH7* (ICGi022-A + *MYH7* p.M659I), and iPSC line from the patient with the variant (ICGi019-B) with antibodies to sarcomeric α-actinin. Scale bar—50 µm. (**b**) Comparison of cardiomyocyte areas for the iPSC lines from healthy donors and with p.M659I (c.1977G > A) mutation in *MYH7* (introduced with CRISPR/Cas9 or patient-specific). (**c**) Comparison of diastolic calcium level in cardiomyocytes derived from the iPSC lines of healthy donors and with p.M659I (c.1977G > A) mutation in *MYH7* (introduced with CRISPR/Cas9 or patient-specific).

One of the most common HCM features in iPSC-based models of the disease is an enlarged size of cardiomyocytes [5,6,7,9,10,30]. The cardiomyocytes derived from the iPSCs with the introduced p.M659I (c.1977G > A) mutation in *MYH7*, patient-specific iPSCs, and iPSCs from healthy donors were visualized by immunofluorescence staining with antibodies to sarcomeric α-actinin, a cardiomyocyte-specific marker (Figure 3a). The calculation of iPSC-derived cardiomyocyte areas showed that the cardiomyocytes carried the p.M659I (c.1977G > A) mutation in *MYH7* (introduced with CRISPR/Cas9 or patient-specific) had an increased size in comparison with those differentiated from the iPSCs of healthy donors (Figure 3b).

Another feature that was revealed in iPSC-based HCM models and was supposed to precede cardiomyocyte hypertrophy is elevated intracellular diastolic calcium level [5,6,9,15,19,30,31]. A calcium-dependent fluorescent dye, Fluo-8 AM, was used to compare diastolic calcium levels in the cardiomyocytes derived from the iPSCs with the introduced p.M659I (c.1977G > A) mutation in *MYH7*, patient-specific iPSCs, and iPSCs from healthy donors. In the cardiomyocytes that carried the p.M659I (c.1977G > A) mutation in *MYH7* (introduced with CRISPR/Cas9 or patient-specific), the diastolic calcium level was demonstrated to be elevated compared to those differentiated from the iPSCs of healthy donors (Figure 3c).

**Figure 4:**
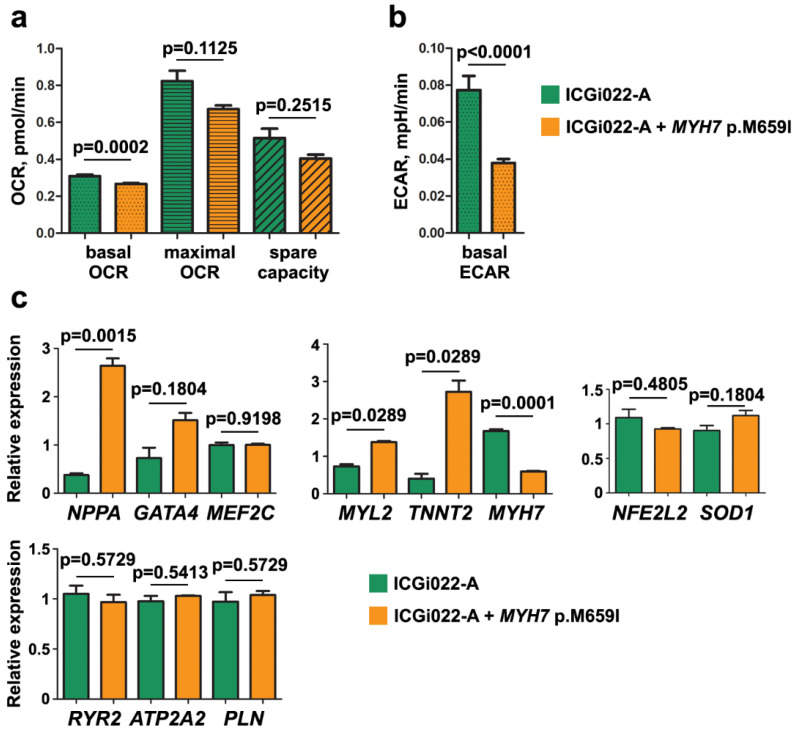
Cardiomyocytes derived from the iPSC line with introduced p.M659I (c.1977G > A) mutation in *MYH7* demonstrate disrupted energy metabolism and changes in expression pattern of HCM-related genes. (**a**) Comparison of oxygen consumption rate (OCR) in cardiomyocytes derived from the iPSC line with introduced p.M659I (c.1977G > A) mutation in *MYH7* (ICGi022-A + *MYH7* p.M659I) and the ICGi022-A iPSC line used for *MYH7* editing. (**b**) Comparison of extracellular acidification rate (ECAR) in cardiomyocytes derived from the iPSC line with introduced p.M659I (c.1977G > A) mutation in *MYH7* (ICGi022-A + *MYH7* p.M659I) and the ICGi022-A iPSC line used for *MYH7* editing. (**c**) Comparison of gene expression level in cardiomyocytes derived from the iPSC line with introduced p.M659I (c.1977G > A) mutation in *MYH7* (ICGi022-A + *MYH7* p.M659I) and the ICGi022-A iPSC line used for *MYH7* editing.

A disruption of mitochondria functioning was also found in some iPSC-based HCM models [14,19,20]. We assessed mitochondrial respiration (oxygen consumption rate, OCR) in cardiomyocytes derived from the iPSC line with the introduced p.M659I (c.1977G > A) mutation in *MYH7* and the original ICGi022-A line (healthy donor) using Seahorse technology (Agilent, Santa Clara, CA, USA). To study mitochondrial respiration, the protocol of cardiac iPSC differentiation was modified. After metabolic selection, cardiomyocytes were cultured for 2 weeks in the presence of 2 µM CHIR99021 and for another 2 weeks in a medium for cardiomyocyte maturation, which contained low glucose and high oxidative substrate concentrations [32]. In iPSC-derived cardiomyocytes with the introduced p.M659I (c.1977G > A) mutation in *MYH7*, basal OCR was significantly lower than in those of the healthy donor (*p*-value = 0.0002). At the same time, no differences in the maximal OCR and spare capacity were revealed (Figure 4a). Extracellular acidification rate (ECAR) reflecting glycolysis level was also investigated. Basal ECAR was decreased in iPSC-derived cardiomyocytes with introduced p.M659I (c.1977G > A) mutation in *MYH7* compared to those of the healthy donor (*p*-value < 0.0001) (Figure 4b).

In the cardiomyocytes derived from the iPSC line with the introduced p.M659I (c.1977G > A) mutation in *MYH7* and the original ICGi022-A line with the maturation stage, the gene expression pattern was also examined by RT-qPCR. The expression levels of well-known HCM-associated genes (*NPPA*, *GATA4*, *MEF2C*) [5,6,30,31], sarcomere genes (*MYH7*, *MYL2*, *TNNT2*) [5,6,7,30], as well as genes involved in the calcium release from the sarcoplasmic reticulum and its re-uptake (*RYR2*, *ATP2A2*, *PLN*) [5,6,7,8,30] and genes of the antioxidant defense system (*NFE2L2*, *SOD1*) [33,34] were compared. Several HCM-related genes (*NPPA*, *MYL2*, *TNNT2*) were shown to be upregulated, whereas *MYH7* was downregulated in the cardiomyocytes with the introduced p.M659I (c.1977G > A) mutation in *MYH7* (Figure 4c). The expression level of *GATA4*, *MEF2C,* and the genes involved in calcium homeostasis regulation and antioxidant defense did not differ significantly between cardiomyocytes derived from the iPSC line with the introduced p.M659I (c.1977G > A) mutation in *MYH7* and the original ICGi022-A line.

Thus, introducing the p.M659I (c.1977G > A) mutation with CRISPR/Cas9 in *MYH7* caused the appearance of HCM traits: increased cardiomyocyte size and diastolic calcium level, changes in the gene expression pattern, and decreased energy metabolism (mitochondrial respiration and glycolysis).

## 3. Discussion

iPSC editing with CRISPR/Cas9 and the investigation of relevant differentiated derivatives of isogenic iPSCs allow for the unraveling of the contribution of genetic variants of unknown significance in disease pathogenesis [11,12,13]. In this study, the approach was applied to establish the clinical significance of the p.M659I (c.1977G > A) variant in *MYH7,* which was found earlier in the genetic screening of HCM patients [23]. The amino acid substitution is located in a protein region that is highly conserved in vertebrates [35]. Sensitivity to amino acid changes was demonstrated by several studies that found pathogenic variants associated with cardiomyopathies in the region [35,36]. In silico analysis suggests that a substitution in close proximity to p.M659I (p.L655M) in β-myosin heavy chain disrupts the actin–myosin interaction, which leads to weaker binding energy and the inefficient functioning of the complex [35]. Rare frequency and all the in silico data indicate the pathogenicity of the variant. However, functional studies are needed to decipher its clinical significance.

In our previous study, we also used the electroporation of CRISPR/Cas9 in the form of ribonucleoprotein complexes and single-stranded donor oligonucleotides with a mutation to the iPSCs of the healthy donor for introducing a variant of unknown significance, p.Asn515del (c.1543_1545delAAC), in *MYBPC3* [37]. All the iPSC clones with the introduced p.Asn515del mutation demonstrated editing events in both alleles of *MYBPC3*: nine iPSC clones carried the p.Asn515del mutation in one allele and indels in the other, whereas three iPSC clones were homozygous at the deletion. Considering the capacity of CRISPR/Cas9 ribonucleoprotein complexes to induce efficiently double-strand breaks in both alleles, we aimed at generating iPSCs heterozygous at the p.M659I (c.1977G > A) variant in *MYH7,* as it occurs in the HCM patient. To achieve the goal, we modified the editing process by using two types of donor oligonucleotides: not only the one that contained the mutation but also the one that corresponded to nucleotide sequence of the healthy donor. However, only one iPSC clone out of 70 (1.43%) demonstrated homologous recombination with both types of donor oligonucleotides, and that clone was heterozygous at the p.M659I (c.1977G > A) mutation in *MYH7*. Thus, two single-stranded donor oligonucleotides (with and without mutation) can be used for generating iPSCs heterozygous at mutations of interest. This may be important for studying HCM and other cardiomyopathies with autosomal dominant inheritance [38]. However, the method needs to be further optimized to increase the efficiency of obtaining iPSCs with desired editing events.

There is a hypothesis suggesting that HCM-causing sarcomeric mutations result in increased myofilament calcium sensitivity, which in turn leads to increased ATP consumption at the sarcomeres and a lack of ATP molecules for other cell processes. One of the processes is ATP-dependent calcium re-uptake to the sarcoplasmic reticulum via ATP2A2. Decreased efficiency of calcium re-uptake results in an elevated diastolic calcium level in cardiomyocytes, which can cause diastolic dysfunction and arrhythmias in HCM [39]. The increased calcium level in cardiomyocytes, in turn, triggers the calcineurin-NFAT signaling pathway that leads to NFAT re-localization to the nucleus and the activation of hypertrophy mediators such as *GATA4* and *MEF2C* [5,6]. In line with this suggestion, we found an increased diastolic calcium level and enlarged size of the cardiomyocytes derived from iPSCs carrying the p.M659I (c.1977G > A) mutation in *MYH7* (introduced with CRISPR/Cas9 or patient-specific) compared to those derived from iPSCs of the healthy donors. The data on gene expression in the cardiomyocytes derived from the iPSC line with the introduced p.M659I (c.1977G > A) mutation in *MYH7* and the original ICGi022-A line of the healthy donor revealed upregulation of natriuretic peptide A (*NPPA*), which was shown to be one of the hypertrophy hallmarks in numerous studies of iPSC-based HCM models [5,6,30,31] and transcriptomes of HCM patients’ heart tissues [40,41,42,43,44]. Interestingly, the expression level of two hypertrophy mediators, *GATA4* and *MEF2C* [6,30], was similar in both types of cardiomyocytes. This suggests that some other mediators may be essential for cardiomyocyte hypertrophy in the case of the p.M659I (c.1977G > A) variant in *MYH7*. The hypertrophy of the cardiomyocytes with the introduced p.M659I (c.1977G > A) mutation in *MYH7* was confirmed not only by *NPPA* upregulation but also by the elevated expression level of two sarcomere genes (*MYL2*, *TNNT2*) that were often upregulated in iPSC-based HCM models [6,7,30]. At the same time, *MYH7* was downregulated in the cardiomyocytes with the introduced p.M659I (c.1977G > A) mutation in *MYH7*. It is worth noting that the expression level of the gene is mutation-specific and can be both upregulated [6,30] and downregulated [5] in iPSC-based HCM models. Despite the increased diastolic calcium level, no differences in the expression pattern of genes involved in calcium homeostasis regulation (*RYR2*, *ATP2A2*, *PLN*) between the cardiomyocytes with the introduced p.M659I (c.1977G > A) mutation in *MYH7* and from the original ICGi022-A line were observed. Previous studies of iPSC-based HCM models revealed that the expression level of the calcium homeostasis genes depended on mutation and could be both upregulated and downregulated or demonstrated no changes between “diseased” and control cardiomyocytes [5,6,7,8,30]. Thus, the enlarged size, the upregulation of a number of HCM-related genes, and the elevated diastolic calcium level that were found in cardiomyocytes derived from the iPSCs with the introduced p.M659I (c.1977G > A) mutation in *MYH7* indicate the pathogenicity of the variant.

Another consequence of calcium accumulation at the sarcomeres is a calcium deficit within the mitochondria, which may inhibit oxidative phosphorylation and ATP production and increase reactive oxygen species (ROS) concentration over time [39]. We decided to investigate mitochondrial respiration in cardiomyocytes derived from the iPSC line with the introduced p.M659I (c.1977G > A) mutation in *MYH7* and the original ICGi022-A line of the healthy donor. However, iPSC-derived cardiomyocytes are characterized by metabolic immaturity and use predominantly glycolysis, but not oxidative phosphorylation [45]. Several studies demonstrated that the oxidative phosphorylation level in iPSC-derived cardiomyocytes might be augmented when cultured in the maturation medium [32,46]. This prompted us to modify the protocol of directed cardiac iPSC differentiation for examining mitochondrial respiration. A stage of cultivation in the medium promoting cardiomyocyte maturation was added. We showed that the basal oxygen consumption rate (OCR) was decreased in cardiomyocytes with the p.M659I (c.1977G > A) mutation in *MYH7* introduced with CRISPR/Cas9 compared to those of the healthy donor. No impact of the p.M659I (c.1977G > A) mutation in *MYH7* on the maximal OCR and spare capacity was observed. Nevertheless, some studies revealed an increase in basal and the maximal OCR in iPSC-derived cardiomyocytes with two other mutations in the β-myosin motor domain and a mutation in cardiac α-actin (*ACTC1*) [14,19,20]. To further verify the energy deficiency theory and appearance of oxidative stress due to ROS accumulation, we also compared the expression level of two genes of antioxidant defense (*NFE2L2* and *SOD1*) [33,34] between the cardiomyocytes derived from the iPSC line with the introduced p.M659I (c.1977G > A) mutation in *MYH7* and the original ICGi022-A line but found no significant differences. This finding is in agreement with previous data on different basal and maximal OCR but similar ROS levels in iPSC-derived cardiomyocytes with and without a pathogenic variant in *MYH7* [14]. Thus, our results support, to some extent, the energy deficiency theory in HCM, but the impact of the p.M659I (c.1977G > A) variant in *MYH7* on the energy metabolism and oxidative stress in cardiomyocytes should be investigated in more detail.

## 4. Materials and Methods

### 4.1. Editing MYH7 with CRISPR/Cas9 in iPSCs of Healthy Donor

Single-guide RNA and donor oligonucleotides were designed using Benchling and IDT (https://www.benchling.com/ and https://www.idtdna.com/, accessed on 13 May 2022) (Table 2). First, 100 pmol of single-guide RNA (Synthego, Redwood City, CA, USA) and 20 pmol of Cas9_NLS (NEB, Ipswich, MA, USA) were incubated for 20 min at room temperature. The ribonucleoprotein complexes, together with 300 ng of 1:1 mixture of two single-stranded donor oligonucleotides with and without the c.1977G > A (p.M659I) mutation (Biolegio, Nijmegen, The Netherlands), were electroporated into 1 × 10^5^ iPSCs of the ICGi022-A line [27] on a Neon Transfection System (Thermo Fisher Scientific, Waltham, MA, USA) using program: 1100 V, 30 ms, 1 time. The electroporated cells were transferred to a feeder layer in an iPSC medium without antibiotics supplemented with 10 ng/mL Y-27632 (Sigma–Aldrich, Darmstadt, Germany). Forty-eight hours later, the cells were subcloned into 96-well plates. iPSC clones were cultured at 37 °C in 5% CO_2_ in the iPSC medium: KnockOut DMEM supplemented with 15% KnockOut Serum Replacement, 0.1 mM MEM Non-Essential Amino Acids Solution, 1× penicillin-streptomycin, 1 mM GlutaMAX (all reagents from Thermo Fisher Scientific, Waltham, MA, USA), 0.05 mM 2-mercaptoethanol (Amresco, Solon, OH, USA), and 10 ng/mL bFGF (SCI-store, Moscow, Russia). iPSC clones were passaged with TrypLE™ Express Enzyme (Thermo Fisher Scientific, Waltham, MA, USA) at a ratio of 1:10 every 4–5 days.

### 4.2. Analysis of Introduced Mutations and CRISPR/Cas9 off-Target Activity

Genomic DNA was isolated from iPSCs using Wizard^®^ Genomic DNA Purification Kit (Promega, Madison, WI, USA). Regions contained Exon 18 of *MYH7* or predicted CRISPR/Cas9 off-target sites were amplified by PCR with BioMaster HS-Taq PCR-Color (2×) (Biolabmix, Novosibirsk, Russia) on a T100 Thermal Cycler (Bio-Rad, Hercules, CA, USA) using program: 95 °C—3 min; 35 cycles: 95 °C—30 s, 62 °C—30 s, 72 °C—30 s; 72 °C—5 min. The primers used are provided in Table 2. Sanger sequencing of PCR products was conducted using Big Dye Terminator V. 3.1. Cycle Sequencing Kit (Applied Biosystems, Austin, TX, USA) and analyzed at the SB RAS Genomics Core Facility (Novosibirsk, Russia).

### 4.3. Spontaneous In Vitro Differentiation

Spontaneous differentiation of iPSCs was performed in embryoid bodies as described earlier [47]. iPSCs were incubated for 40 min with 0.15% Collagenase IV (Thermo Fisher Scientific, Waltham, MA, USA). The suspension was centrifuged for 5 min at 200 g. Cells were transferred on Petri dishes coated with 1% agarose (Helicon) and were cultured for 2 weeks in DMEM/F12 (1:1) medium supplemented with 15% KnockOut Serum Replacement, 0.1 mM MEM Non-Essential Amino Acids Solution, 1× penicillin-streptomycin, 1 mM GlutaMAX (all reagents from Thermo Fisher Scientific, Waltham, MA, USA) for embryoid body formation. Medium was changed every 3 days. The embryoid bodies were plated on 8-well chambered coverglass (Thermo Fisher Scientific, Waltham, MA, USA) coated with Matrigel (Corning, New York, NY, USA) and cultivated for another week under the same conditions.

### 4.4. Immunofluorescence Staining

Cells were fixed for 10 min in 4% paraformaldehyde (Sigma–Aldrich, Darmstadt, Germany), permeabilized for 10 min in 0.4% Triton-X100 (Sigma–Aldrich, Darmstadt, Germany), and incubated for 30 min with 1% bovine serum albumin (VWR, Solon, OH, USA) (all steps were performed at room temperature). Cells were further incubated overnight at 4 °C with primary antibodies and for 1 h at room temperature with secondary antibodies. The antibodies used are provided in Table 2. After each incubation with antibodies, cells were washed twice for 15 min with PBS. Nuclei were counterstained with DAPI (Sigma–Aldrich, Darmstadt, Germany). Images were recorded using a Nikon Eclipse Ti-E and NIS Elements software (Nikon, Tokyo, Japan).

### 4.5. RT-qPCR

RNA was isolated with TRIzol Reagent and purified from possible DNA contaminations using Invitrogen™ DNA-*free*™ DNA Removal Kit (all reagents from Thermo Fisher Scientific, Waltham, MA, USA). Reverse transcription of 1 µg of RNA was performed with the M-MuLV reverse transcriptase (Biolabmix, Novosibirsk, Russia). All procedures were conducted according to the manufacturers’ protocols. RT-qPCR was performed with BioMaster HS-qPCR SYBR Blue 2× (Biolabmix, Novosibirsk, Russia) on a LightCycler 480 System (Roche, Basel, Switzerland) and QuantStudio™ 5 Real-Time PCR System (Applied Biosystems, Life Technologies Holdings Pte. Ltd., Singapore) for gene expression analysis in iPSCs and iPSC-derived cardiomyocytes, respectively, using program: 95 °C—5 min; 40 cycles: 95 °C—10 s, 60 °C—1 min. CT values were normalized to *B2M* using the ΔΔCT method for the pluripotency genes. Data on gene expression in iPSC-derived cardiomyocytes were analyzed in qbase+ V3.4. *B2M* and *GAPDH* were used as reference genes. All the primers are provided in Table 2.

### 4.6. Karyotyping

Cell treatment and metaphase collection were performed as described previously [48]. iPSCs were plated at a ratio of 1:4 on four wells of 12-well plate 48 h before metaphase collection. iPSC medium was changed to the fresh one and different concentrations of Colcemide (from 25 to 50 ng/mL) were added 2.5 h before metaphase collection. Cells were disaggregated with TrypLE™ Express Enzyme (Thermo Fisher Scientific, Waltham, MA, USA). Hypotonic treatment was performed for 20 min at 37 °C in 0.28% KCl. Cells were fixed in the Carnoy’s solution (methanol–acetic acid 3:1). Karyotype of the iPSC clone was analyzed at the Tomsk National Research Medical Center of the Russian Academy of Sciences using a Zeiss Axio Scope.A1 (Zeiss, Oberkochen, Germany) and Ikaros KARyOtyping Software (MetaSystems, Altlussheim, Germany). Fifty metaphase plates were analyzed.

### 4.7. Mycoplasma Detection

Mycoplasma contamination of the iPSC clone was detected by PCR with BioMaster HS-Taq PCR-Color (2×) (Biolabmix, Novosibirsk, Russia) on a T100 Thermal Cycler (Bio-Rad, Hercules, CA, USA) using program: 95 °C—3 min; 35 cycles: 95 °C—15 s, 67 °C—15 s, 72 °C—20 s; 72 °C—5 min. The primers used are listed in Table 2.

### 4.8. Directed iPSC Differentiation into Cardiomyocytes

iPSCs were cultured for two-to-three passages in Essential 8 Medium (Thermo Fisher Scientific, Waltham, MA, USA) on wells coated with Matrigel (Corning, New York, NY, USA). iPSCs were plated at a ratio of 1:6–1:10 on Matrigel-coated 12-well plates. After cells reached 80–90% confluency, RPMI 1640 medium supplemented with 1× penicillin-streptomycin, 1× B27 Supplement minus insulin (all reagents from Thermo Fisher Scientific, Waltham, MA, USA), and 6 µM CHIR99021 (Sigma–Aldrich, Darmstadt, Germany) was added for 48 h to induce differentiation. Seventy-two hours after, RPMI 1640 medium supplemented with 1× penicillin-streptomycin, 1× B27 Supplement minus insulin, and 5 µM IWP2 (Sigma–Aldrich, Darmstadt, Germany) or 2 µM Wnt-C59 (Selleck Chemicals, Houston, TX, USA), was added for another 48 h. Starting from Day 7 of differentiation, cells were cultivated in RPMI 1640 medium supplemented with 1× penicillin-streptomycin and 1× B27 Supplement (Thermo Fisher Scientific, Waltham, MA, USA) (RPMI/B27 medium). On Day 9 of differentiation, cells were subjected to metabolic selection for 7–9 days in RPMI 1640 medium without D-glucose (Thermo Fisher Scientific, Waltham, MA, USA) supplemented with 1× penicillin-streptomycin, 213 µg/mL L-ascorbic acid 2-phosphate sesquimagnesium salt hydrate (Sigma–Aldrich, Darmstadt, Germany), 500 µg/mL bovine serum albumin (VWR, Solon, OH, USA), and 5 mM sodium DL-lactate (Sigma–Aldrich, Darmstadt, Germany) [29]. Purified cardiomyocytes were dissociated with 0.25% trypsin, plated on Matrigel-coated wells, and cultivated in the RPMI/B27 medium.

### 4.9. Analysis of Cardiomyocyte Size

To define cardiomyocyte boundaries, immunofluorescence staining with antibodies to sarcomeric α-actinin (Table 2) was performed as described in the Immunofluorescence Staining Section (Section 4.4). Images of cardiomyocytes in random fields of view were used to calculate cell surfaces in ImageJ 1.53k. No less than 100 cardiomyocytes were analyzed for each iPSC line.

### 4.10. Calcium Imaging

iPSC-derived cardiomyocytes were incubated in the RPMI/B27 medium with 4 µg/mL Fluo-8 AM (Abcam, Cambridge, UK) for 30 min in a CO_2_ incubator (Binder, Tuttlingen, Germany). The medium was then replaced with Tyrode’s solution (140 mM NaCl, 4.5 mM KCl, 10 mM glucose, 10 mM HEPES, 1 mM MgCl_2_, 1.8 mM CaCl_2_, and 1% penicillin-streptomycin, pH 7.4) (Sigma–Aldrich, Darmstadt, Germany) that was warmed to 37 °C. Calcium-dependent fluorescence was recorded for 10 s at 100 ms exposure on a Nikon Eclipse Ti-E with NIS Elements software (Nikon, Tokyo, Japan). To determine diastolic calcium-dependent fluorescence, the videos were processed with ImageJ 1.53k, including subtraction of background fluorescence noise.

### 4.11. Seahorse

After metabolic selection, cardiomyocytes were plated on Matrigel-coated 6-wells and cultured for 2 weeks in the RPMI/B27 medium supplemented with 2 µM CHIR99021 (Sigma–Aldrich, Darmstadt, Germany). On Day 30 of differentiation, cardiomyocytes were plated on Matrigel-coated 12-wells at a ratio of 8.5–9.5 × 10^5^ cells per well and cultivated for 2 weeks in the cardiomyocyte maturation medium [32]: DMEM with L-glutamine but without D-glucose (Thermo Fisher Scientific, Waltham, MA, USA) supplemented with 3 mM glucose, 10 mM L-lactate, 5 mM Creatine monohydrate, 2 mM L-carnitine, 0.5 mM L-ascorbic acid 2-phosphate sesquimagnesium salt hydrate (all reagents from Sigma–Aldrich, Darmstadt, Germany), 5 µg/mL Vitamin B12, 0.82 µM Biotin, 2 mM Taurine (all reagents from HiMedia, Thane, India), 0.1 mM MEM Non-Essential Amino Acids Solution, 1× B27 Supplement, 1% KoSR, 1× penicillin-streptomycin, and 0.5% (*w*/*v*) Albumax (all reagents from Thermo Fisher Scientific, Waltham, MA, USA).

On Day 45 of differentiation, cardiomyocytes were plated on Matrigel-coated Seahorse XFp Cell Culture Miniplates (Agilent, Santa Clara, CA, USA) at a ratio of 4 × 10^4^ cells per well and were cultured for a week in the cardiomyocyte maturation medium. Oxygen consumption rate (OCR) and extracellular acidification rate (ECAR) were measured on a Seahorse XF HS Mini Analyzer using Seahorse XFp Cell Mito Stress Test Kit (Agilent, Santa Clara, CA, USA) with 1.5 µM oligomycin, 2 µM FCCP, and 0.5 µM rotenone/antimycin A according to the manufacturer’s protocols. The data obtained were analyzed in Wave 2.6.3 (Agilent, Santa Clara, CA, USA) and were normalized to average cell number in five random 20× fields of view for each well. Cardiomyocytes were visualized using immunofluorescence staining with antibodies to sarcomeric α-actinin on a Nikon Eclipse Ti-E with NIS Elements software (Nikon, Tokyo, Japan).

### 4.12. Statistics

Statistical significance of differences between iPSC-derived cardiomyocytes in size and diastolic calcium level was determined using one-way ANOVA with Tukey’s correction for multiple comparisons and Kruskal–Wallis test with Dunn’s test for multiple comparisons, respectively. Differences between iPSC-derived cardiomyocytes in OCR, ECAR, and gene expression level were verified by Mann–Whitney U test. *p*-values < 0.05 were considered to be significant. *p*-values for gene expression level in iPSC-derived cardiomyocytes were calculated in qbase+ V3.4. The other calculations, box plots, and column bar graphs were conducted in GraphPad Prism Version 5.00 for Windows. The data are presented as mean with minimal and maximal values and mean ± SEM for box plots and column bar graphs, respectively.

## 5. Conclusions

Using CRISPR/Cas9, a variant of unknown significance, p.M659I (c.1977G > A) in *MYH7*, was introduced into iPSCs of a healthy donor. The application of two single-stranded donor oligonucleotides (with and without the p.M659I (c.1977G > A) mutation in *MYH7*) allowed for the generation of an iPSC line heterozygous at the substitution. No CRISPR/Cas9 off-target activity was revealed. Despite *MYH7* editing, the iPSC line retained pluripotent state and normal karyotype. Cardiomyocytes derived from the iPSC line with the introduced p.M659I (c.1977G > A) mutation in *MYH7* reproduced characteristic HCM traits such as enlarged size, changes in the expression level of HCM-related genes, elevated diastolic calcium level, and some abnormalities in energy metabolism compared to those derived from the original iPSC line. These findings are in agreement with population and in silico data and strongly support the pathogenicity of the variant. This work is a first attempt of functional studies for clarifying the clinical significance of the p.M659I (c.1977G > A) variant in *MYH7*, which may be important for the interpretation of the genetic screening data of HCM patients. In addition, the isogenic iPSC lines generated in the work may be used for examining the molecular mechanisms of the disease triggered by the p.M659I (c.1977G > A) variant in *MYH7,* especially the way how the variant may influence energy metabolism and oxidative stress in cardiomyocytes.

## Figures and Tables

**Table 1:** Prediction for pathogenicity of p.M659I (c.1977G > A) variant in *MYH7* based on the data of in silico analysis [23].

Tool	Score	Prediction
SIFT	0.001	Deleterious
PolyPhen2 HDIV	0.957	Damaging
PolyPhen2 HVAR	0.996	Damaging
MutationTaster	1	Deleterious
MutationAssessor	2.815	Medium deleterious probability
FATHMM	−3.74	Deleterious
PROVEAN	−3.38	Deleterious
fathmm-MKL	0.992	Deleterious
MetaSVM	1.069	Deleterious
MetaLR	0.935	Deleterious

**Table 2:** Oligonucleotides and antibodies used in the study.

Oligonucleotides			
	Gene/Locus	Product Size	Nucleotide Sequence (5′-3′)
Protospacer for introducing p.M659I (c.1977G > A) mutation	*MYH7*, Exon 18	20 b	GGGATGGGTGGAGCGCAAGT
Donor oligonucleotide with p.M659I (c.1977G > A) mutation	*MYH7*, Exon 18	89 b	TATTGCATTTTTGGCCACAGGAAAATCTGAACAAGCTGATAACAAACTTGCGCTCCACCCATCCCCACTTTGTACGTTGTATCATCCCT
Donor oligonucleotide without p.M659I (c.1977G > A) mutation	*MYH7*, Exon 18	89 b	TATTGCATTTTTGGCCACAGGAAAATCTGAACAAGCTGATGACAAACTTGCGCTCCACCCATCCCCACTTTGTACGTTGTATCATCCCT
Mutation analysis	*MYH7*, Exon 18	258 bp	TCCTTCCTTCTTCTCCTCTCTT/GTGGTGGTAGGTAGGGAGAT
CRISPR/Cas9 off-target activity analysis	chr4:14217409-14217428	559 bp	TCTGGTAAGAGCCTGACTTCTG/TCCCACCTGCCATTGGAATA
	chr7:57819587-57819606	378 bp	ACGATACTCAAGGCCCAATCT/TGGTGTTTCCTCATCCTGGT
	chr8:139343032-139343051	535 bp	GCCAGGAAAGTTCAGTGGTTAG/CCCTCTCTCTTCCTGCTCTTAT
	chr20:9996001-9996020	575 bp	GACTTGTAATAACTCTCACTCACCTAAA/CCAGGCAATGTTAAGCCTTCAT
	chr2:241709069-241709088	541 bp	TCCCGTGTGGATTTCTTTAGGT/TGTAGGCGTTCTGGATCTTCTG
Mycoplasma detection	16S ribosomal RNA gene	280 bp	GGGAGCAAACAGGATTAGATACCCT/TGCACCATCTGTCACTCTGTTAACCTC
Reference genes (RT-qPCR)	*B2M*	90 bp	TAGCTGTGCTCGCGCTACT/TCTCTGCTGGATGACGTGAG
	*GAPDH*	202 bp	TGTTGCCATCAATGACCCCTT/CTCCACGACGTACTCAGCG
Pluripotency markers (RT-qPCR)	*OCT4*	144 bp	GGGAGATTGATAACTGGTGTGTT/GTGTATATCCCAGGGTGATCCTC
	*NANOG*	116 bp	TTTGTGGGCCTGAAGAAAACT/AGGGCTGTCCTGAATAAGCAG
	*SOX2*	100 bp	GCTTAGCCTCGTCGATGAAC/AACCCCAAGATGCACAACTC
HCM-associated genes (RT-qPCR)	*NPPA*	95 bp	GATAACAGCCAGGGAGGACAAG/CAAGATGACACAAATGCAGCAGAG
	*GATA4*	99 bp	CCTGTGAGTTGGAGACTTCTTT/CCTCGGTGCTAGAAACACAA
	*MEF2C*	117 bp	CTGGTCTCACCTGGTAACTTGAAC/CTTGCTGCCTGGTGGAATAAGA
Sarcomere genes (RT-qPCR)	*MYH7*	83 bp	TCGTGCCTGATGACAAACAGGAGT/ATACTCGGTCTCGGCAGTGACTTT
	*MYL2*	93 bp	GGACCCTGAGGAAACCATTCT/GTCAGCATTTCCCGAACGTAATC
	*TNNT2*	89 bp	GGCCATTGACCACCTGAATGA/CGAACTTCTCTGCCTCCAAGTTATAG
Calcium homeostasis regulation genes (RT-qPCR)	*RYR2*	86 bp	GTTGCTCCATCGGCAGTATGA/CCTCCACGGACACACCATTTAT
	*ATP2A2*	89 bp	CCACGAGCTGTCAACCAAGATA/GTTGCTACCACCACTCCCATAG
	*PLN*	98 bp	GCTGCCAAGGCTACCTAAA/CAGGACAGGAAGTCTGAAGTTT
Antioxidant defense genes (RT-qPCR)	*NFE2L2*	125 bp	TCTGCCAACTACTCCCAGGT/AACGTAGCCGAAGAAACCTCA
	*SOD1*	194 bp	CTAGCGAGTTATGGCGACGA/CTGCACTGGTACAGCCTGC
**Antibodies**			
	**Antibody**	**Dilution**	**Company, Cat #, and RRID**
Pluripotency markers	Mouse IgG2b anti-OCT3/4	1:50	Santa Cruz Biotechnology, Dallas, TX, USA, Cat # sc-5279, RRID:AB_628051
	Rabbit IgG anti-NANOG	1:200	ReproCELL, Yokohama, Japan, Cat # RCAB003P, RRID: AB_2714012
	Rabbit IgG anti-SOX2	1:200	Cell Signaling Technology, Danvers, MA, USA, Cat # 3579, RRID:AB_2195767
	Mouse IgM anti-TRA-1-60	1:200	Abcam, Cambridge, UK, Cat # ab16288, RRID:AB_778563
Markers of differentiated derivatives	Mouse IgG2a anti-TUBB3	1:500	BioLegend, San Diego, CA, USA, Cat # 801201, RRID:AB_2313773
	Mouse IgG2a anti-αSMA	1:100	Dako, Glostrup, Denmark, Cat # M0851, RRID:AB_2223500
	Mouse IgG1 anti-CK18	1:100	Abcam, Cambridge, UK, Cat # ab668, RRID:AB_305647
Cardiomyocyte markers	Mouse IgG1 anti-sarcomeric α-actinin	1:200	Abcam, Cambridge, UK, Cat # ab9465, RRID:AB_307264
Secondary antibodies	Goat anti-Mouse IgG (H + L) Secondary Antibody, Alexa Fluor 568	1:400	Thermo Fisher Scientific, Waltham, MA, USA, Cat # A11031, RRID:AB_144696
	Goat anti-Rabbit IgG (H + L) Highly Cross-Adsorbed Secondary Antibody, Alexa Fluor 488	1:400	Thermo Fisher Scientific, Waltham, MA, USA, Cat # A11008, RRID:AB_143165
	Goat anti-Mouse IgM Heavy Chain Cross-Adsorbed Secondary Antibody, Alexa Fluor 568	1:400	Thermo Fisher Scientific, Waltham, MA, USA, Cat # A21043, RRID:AB_2535712
	Goat anti-Mouse IgG2a Cross-Adsorbed Secondary Antibody, Alexa Fluor™ 568	1:400	Thermo Fisher Scientific, Waltham, MA, USA, Cat # A21134, RRID:AB_2535773
	Goat anti-Mouse IgG1 Cross-Adsorbed Secondary Antibody, Alexa Fluor™ 488	1:400	Thermo Fisher Scientific, Waltham, MA, USA, Cat # A21121, RRID:AB_2535764

## Data Availability

The data on the iPSC line characterization are available in the Human Pluripotent Stem Cell Registry (https://hpscreg.eu/cell-line/ICGi022-A-8, accessed on 19 December 2023).

## Supplementary Materials

The following supporting information can be downloaded at: https://www.mdpi.com/article/10.3390/ijms25168695/s1.
