# Supplementary material for: Studying Pathogenetic Contribution of a Variant of Unknown Significance, p.M659I (c.1977G > A) in MYH7, to the Development of Hypertrophic Cardiomyopathy Using CRISPR/Cas9-Engineered Isogenic Induced Pluripotent Stem Cells
Source: Int J Mol Sci. 2024 Aug 9;25(16):8695. doi: 10.3390/ijms25168695 (PMC11354791; doi:10.3390/ijms25168695)
Supplement: Supplementary file 1 [file ijms-25-08695-s001.zip › ijms-3102733-supplementary.pdf]

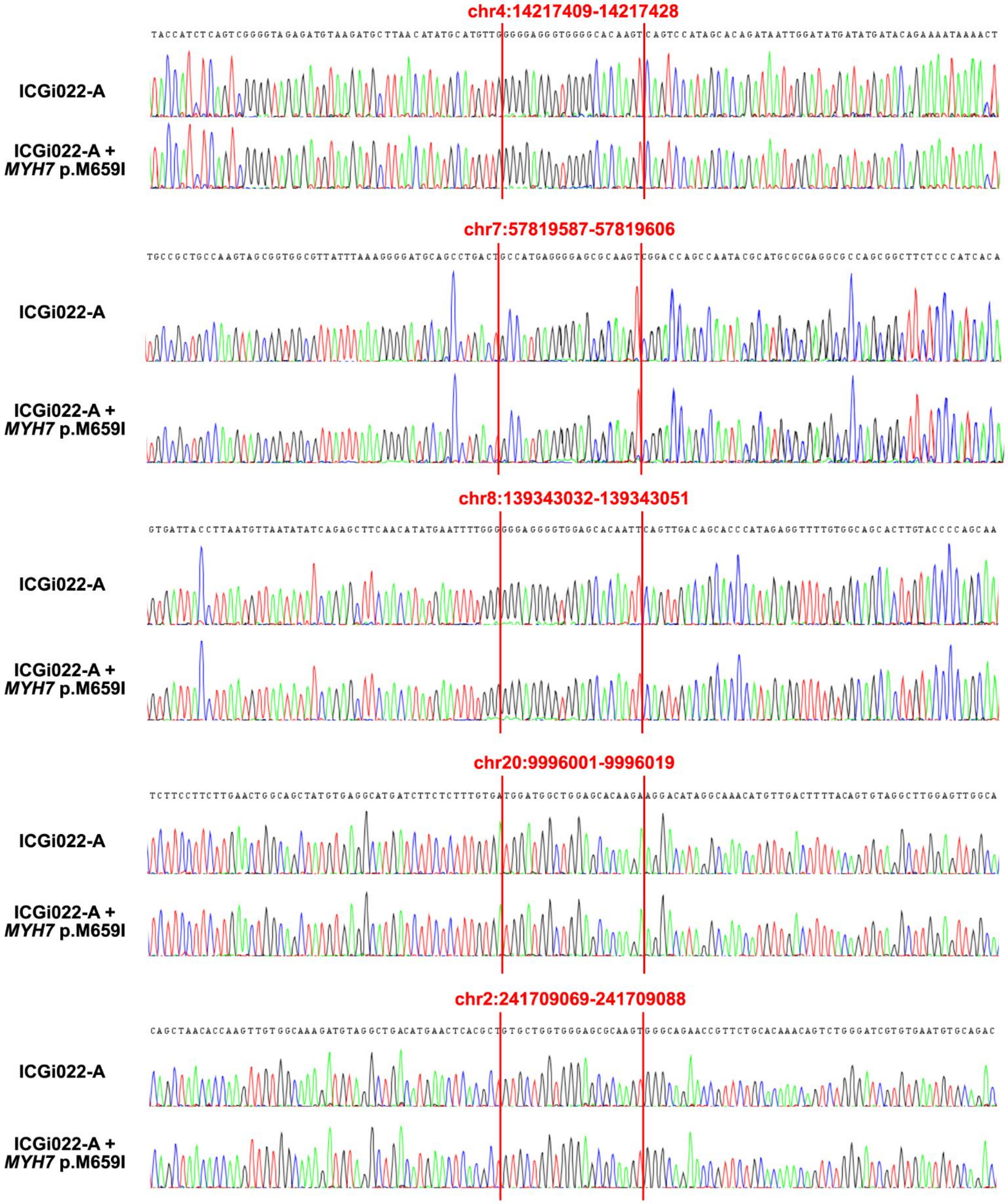

Figure S1. Analysis of CRISPR/Cas9 off-target activity in the iPSC line with introduced p.M659I (c.1977G>A) mutation in *MYH7*. CRISPR/Cas9 off-target sites and their positions in the human genome (hg38) are shown in red. Nucleotide sequences of the CRISPR/Cas9 off-target sites and their surrounding are given for the iPSC line with introduced p.M659I (c.1977G>A) mutation in *MYH7* (ICGi022-A + *MYH7* p.M659I) and ICGi022-A iPSC line used for *MYH7* editing.
